# γ-Glutamyltransferase Variability and the Risk of Mortality, Myocardial Infarction, and Stroke: A Nationwide Population-Based Cohort Study

**DOI:** 10.3390/jcm8060832

**Published:** 2019-06-12

**Authors:** Hye Soo Chung, Ji Sung Lee, Jung A. Kim, Eun Roh, You Bin Lee, So Hyeon Hong, Hye Jin Yoo, Sei Hyun Baik, Nan Hee Kim, Ji A Seo, Sin Gon Kim, Nam Hoon Kim, Kyung Mook Choi

**Affiliations:** 1 Division of Endocrinology and Metabolism, Department of Internal Medicine, Kangnam Sacred Heart Hospital, College of Medicine, Hallym University, #1, Singil-ro, Yeongdeungpo-gu, Seoul 07441, Korea; soo3802@hanmail.net; 2 Clinical Research Center, Asan Medical Center, College of Medicine, Ulsan University, #88, Olympic-ro 43-gil, Songpa-gu, Seoul 05505, Korea; totoro96a@gmail.com; 3 Division of Endocrinology and Metabolism, Department of Internal Medicine, Korea University Guro Hospital, College of Medicine, Korea University, 80 Guro-Dong, Guro-Gu, Seoul 08308, Korea; frances.junga@gmail.com (J.A.K.); roheun@gmail.com (E.R.); yb.snowyday1224@gmail.com (Y.B.L.); kicara@gmail.com (S.H.H.); deisy21@naver.com (H.J.Y.); 103hyun@korea.ac.kr (S.H.B.); 4 Division of Endocrinology and Metabolism, Department of Internal Medicine, Korea University Ansan Hospital, College of Medicine, Korea University, #123, Jeokgeum-ro, Danwon-gu, Ansan-si 15355, Korea; nhkendo@gmail.com (N.H.K.); seo-ji-a@hanmail.net (J.A.S.); 5 Division of Endocrinology and Metabolism, Department of Internal Medicine, Korea University Anam Hospital, College of Medicine, Korea University, #145, Anam-ro, Seongbuk-gu, Seoul 02841, Korea; k50367@korea.ac.kr (S.G.K.); pourlife@naver.com (N.H.K.)

**Keywords:** γ-glutamyltransferase, variability, mortality, myocardial infarction, stroke

## Abstract

Although it has been suggested that the γ-glutamyltransferase (GGT) level is an indicator of cardiometabolic disorders, there is no previous study to evaluate the implication of GGT variability on the development of myocardial infarction (MI), stroke, all-cause mortality, and cardiovascular disease (CVD)-related mortality. GGT variability was measured as the coefficient variance (GGT-CV), standard deviation (GGT-SD), and variability independent of the mean (GGT-VIM). Using the population-based Korean National Health Insurance Service-Health Screening Cohort, we followed 158,736 Korean adults over a median duration of 8.4 years. In multivariable Cox proportional hazard analysis, the risk of mortality, MI, and stroke showed a stepwise increase according to the quartiles of GGT-CV, GGT-SD or GGT-VIM. In the highest quartile of GGT-CV compared to the lowest quartile after adjusting for confounding variables including mean GGT, the hazard ratios (HRs) for incident MI, stroke, mortality, and CVD-related mortality were 1.19 (95% confidence interval (CI), 1.06–1.34; *p* < 0.001), 1.20 (95% CI, 1.10–1.32; *p* < 0.001), 1.41 (95% CI, 1.33–1.51; *p* < 0.001), and 1.52 (95% CI, 1.30–1.78; *p* < 0.001), respectively, which were similar or even higher compared with those associated with total cholesterol variability. This is the first study to demonstrate that high GGT variability is associated with increased risk of MI, stroke, all-cause mortality, and CVD-related mortality in the general population.

## 1. Introduction

Recent studies have reported that the variability of cardiometabolic risk factors such as blood pressure [1,2], glucose [3,4,5,6], and cholesterol [7] is independently associated with the development of cardiovascular diseases (CVDs) or mortality. Hsieh et al. demonstrated that the visit-to-visit variability of blood pressure independently predicted all-cause mortality in patients with type 2 diabetes [1]. Furthermore, systolic blood pressure variability was positively related with risks of major macrovascular events, including myocardial infarction (MI), stroke, or cardiovascular death, in the Action in Diabetes and Vascular Disease: Preterax and Diamicron Modified-Release Controlled Evaluation (ADVANCE) trial [2]. Using data from the ADVANCE trial, Hirakawa et al. found that visit-to-visit variability in HbA1c and fasting glucose was strongly associated with vascular events in type 2 diabetes [6]. Bangalore et al. demonstrated that greater low-density lipoprotein-cholesterol (LDL-C) variability was a strong predictor of MI, stroke, and death, independent of statin dose and achieved LDL-C levels in the Treating-to-New-Targets (TNT) trial [8]. Kim et al. recently reported that high visit-to-visit variability in cholesterol levels is associated with the risk of mortality, MI, and stroke in the general population, using the Korean National Insurance System cohort [7].

γ-glutamyltransferase (GGT) has been established not only as an indicator of potential liver disease but also as a biomarker of CVD outcomes [9]. Additionally, it has been suggested that an elevated GGT level is linked to an increased risk of various diseases, including type 2 diabetes, metabolic syndrome, and mortality [10,11,12]. Longitudinal studies have reported that increased GGT levels are associated with an increased risk of mortality with/without ischemic heart disease [13,14]. Recently, we reported that liver enzymes, especially GGT, are strong predictors for the risk of 1-year mortality after MI or stroke as well as for the long-term risk of MI, stroke, and mortality in the Korean population [15].

However, to the best of our knowledge, no studies have reported the relationship between long-term GGT variability with mortality and CVD. In the present study, we examined the association between visit-to-visit GGT variability and the incidence of mortality, MI, and stroke using the longitudinal National Health Insurance Service-National Health Screening Cohort (NHIS-HEALS) database.

## 2. Experimental Section

### 2.1. Study Participants

**Figure 1:**
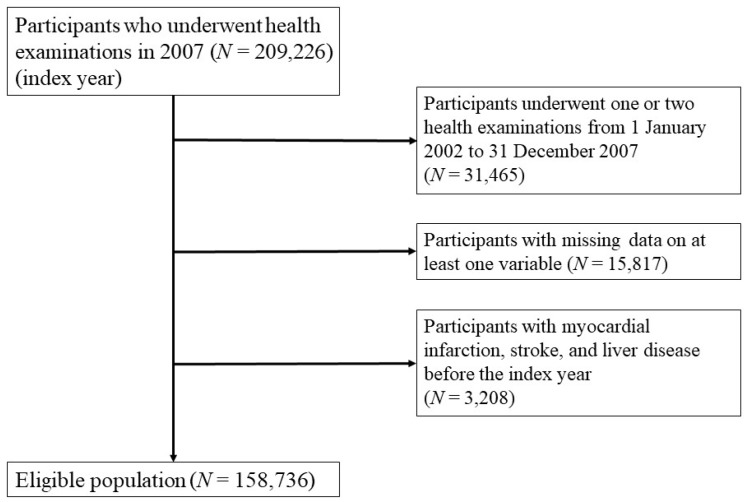
Flow chart of the study population.

The NHIS in Korea is a government-operated mandatory social health insurance program that covers almost the entire (approximately 98%) Korean population recommended to undergo a standardized health examination biannually [16,17]. The NHIS consists of an eligibility database (e.g., age, sex, and socioeconomic variables), a health examination database (questionnaires on health-related behavioral variables and results of laboratory measurements), and a medical history database (diagnosis, medication, admission, and death). Anthropometric and laboratory measurements were examined after an overnight fast, and quality control procedures were checked by the Korean Association of Laboratory Quality Control. The NHIS-HEALS database was used to randomly select approximately 10% of the entire participants within the NHIS database, who were aged from 40 to 79 years. This study was conducted using the participant data of those who had undergone the national health examination in 2007 (index year), and three or more health examinations from 1 January 2002 to 31 December 2007. After excluding individuals who had missing data for at least one variable and those who had a previous diagnosis of MI, stroke, and liver disease prior to 2007, a total of 158,736 individuals were finally included in the analysis (Figure 1). These protocols were approved by the NHIS review committee and the Korea University institutional review board (IRB) approved the study protocol in accordance with the Declaration of Helsinki of the World Medical Association (approval number: KUGH16118-001). Informed consent was waived because anonymous and de-identified information was used for analysis.

### 2.2. Measurements and Definitions

Body mass index (BMI) was calculated as weight in kilograms (kg) divided by the square of height in meters (meters^2^). Smoking status and information concerning alcohol consumption were obtained from a questionnaire undertaken during the health examination. Regular exercise was defined as strenuous physical activity for at least 20 min and ≥5 times/week. Income levels were dichotomized at the lower 10%. The presence of diabetes was defined based on the criteria of a fasting glucose level ≥7.0 mmol/L or the presence of at least one claim per year for the prescription of antidiabetic medication under the International Classification of Diseases, Tenth Revision (ICD-10) codes (E10–E14). The presence of hypertension was defined based on the criteria of systolic/diastolic blood pressure ≥140/90 mmHg or the presence of at least one claim per year for the prescription of an antihypertensive agent under ICD-10 codes (I10–I15). The presence of dyslipidemia was defined based on the criteria of total cholesterol ≥6.2 mmol/L or the presence of at least one claim per year for the prescription of an antihyperlipidemic agent under ICD-10 codes (E78). Liver diseases were defined based on ICD-10 codes, such as malignant neoplasm of the liver and intrahepatic bile ducts (C22), fibrosis and cirrhosis of the liver (K74), and chronic viral hepatitis (B18).

### 2.3. Definition of GGT Variability

GGT variability was determined from at least three measurements of GGT values within-participant on health examinations: 3 measurements (*n* = 79,986, 50.4%), 4 measurements (*n* = 17,178, 10.8%), 5 measurements (*n* = 22,934, 14.4%), and 6 measurements (*n* = 38,638, 24.3%). For descriptive GGT variability, we used three indices of variability, namely, coefficient of variation (GGT-CV), standard deviation (GGT-SD), and variability independent of the mean (GGT-VIM). The CV was defined as the SD divided by the mean (SD/mean) × 100 (%) and VIM was defined as 100× SD/Mean*β*, where *β* is the regression coefficient, based on the natural logarithm of the SD divided by the natural logarithm of the mean.

### 2.4. Study Outcomes

We examined newly occurred MI, stroke, all-cause mortality, and CVD-related mortality as primary outcomes from 1 January 2008 to 31 December 2015. Diagnosis of MI was defined using the International Classification of Diseases, Tenth Revision (ICD-10) codes (I21–I22) during hospitalisation, or these codes having been recorded at least twice. Diagnosis of stroke was based on ICD-10 codes (I63–I64) on admission records and using computed tomography or magnetic resonance imaging claim data. All-cause mortality and CVD-related mortality (ICD 10 codes: I10~I99) were obtained from government databases.

### 2.5. Statistical Analysis

Baseline characteristics are presented as the means ± standard deviations for continuous variables or percentages for categorical variables. Participants were classified into four groups according to the GGT variability quartile. Differences between groups were identified using the analysis of variance (ANOVA) for continuous variables or the *χ*^2^-test for categorical variables. Kaplan–Meier curves for disease-free probability for MI and stroke and survival were obtained for the four groups which were classified and expressed as the quartiles of GGT variability. Hazard ratios (HRs) and 95% confidence interval (CI) values for mortality or cardiovascular diseases were analyzed using Cox proportional hazards models for quartile groups of GGT variability, adjusted for age, sex, BMI, alcohol consumption, smoking, regular exercise, income, diabetes mellitus, hypertension, dyslipidemia, and mean GGT. We tested the assumption of the proportionality of hazards using the numerical method proposed by Lin et al., derived from cumulative sums of martingale-based residuals [18]. We found no evidence of violating the proportional hazards assumption. All statistical results were analyzed using SAS 9.4 (SAS Institute Inc., Cary, NC, USA) and a *p* value < 0.05 was assumed to indicate statistical significance. All statistical analyses were performed by an experienced professional statistician, who was also one of authors.

## 3. Results

### 3.1. Baseline Characteristics of the Study Population

Table 1 shows the baseline characteristics of the study participants according to the quartiles of CV for GGT variability. Higher quartile groups of GGT variability comprised a higher prevalence of current smokers and consumers of alcohol than the lower quartile group. Moreover, the prevalence of metabolic disease factors, such as hypertension, diabetes mellitus, and dyslipidemia, also increased incrementally according to the quartiles of GGT variability. Systolic blood pressure, diastolic blood pressure, and fasting glucose level also increased in the higher quartile groups of GGT variability. Similar relationships of baseline characteristics are shown according to the quartiles of GGT-SD (Table S1) and GGT-VIM (Table S2).

### 3.2. Implications of GGT Variability with All-Cause Mortality, MI, and Stroke

**Figure 2:**
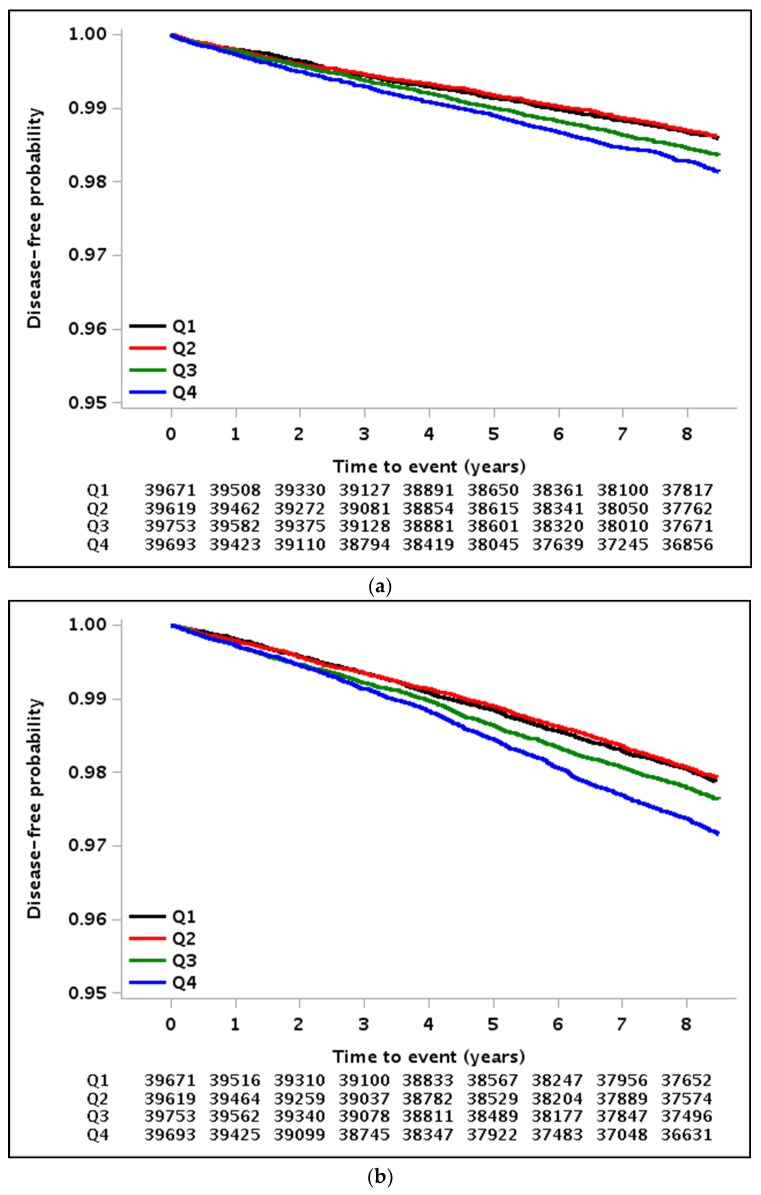

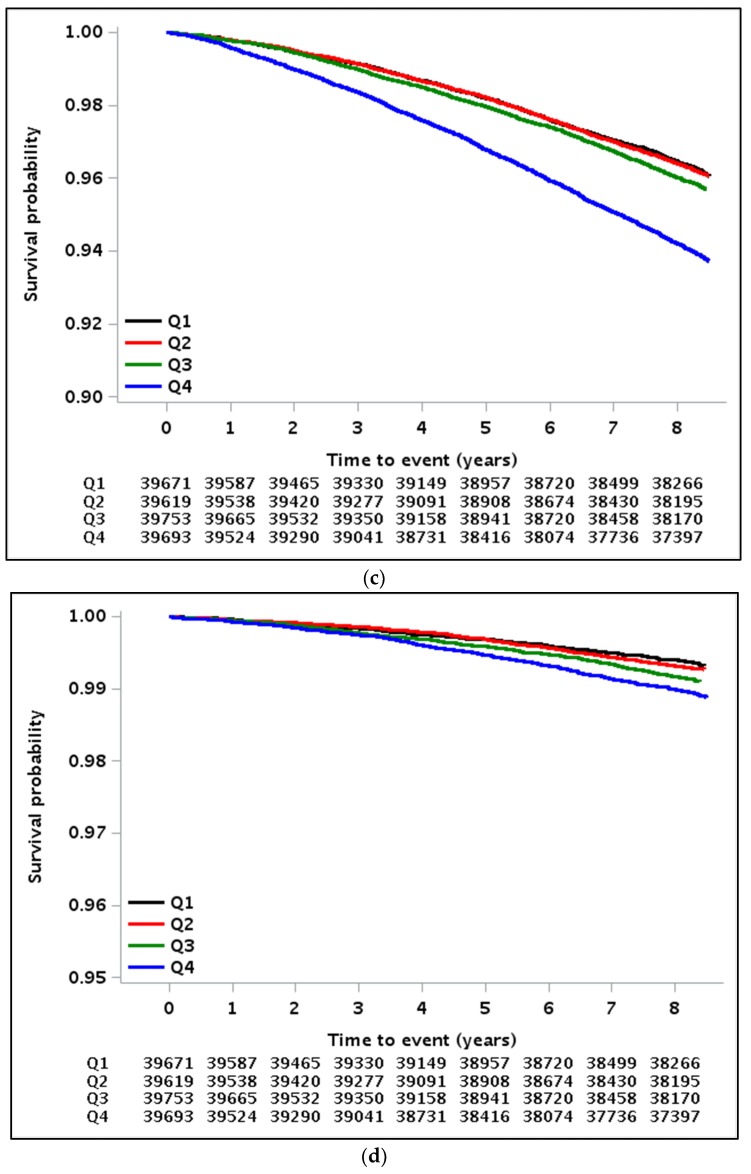
Kaplan–Meier estimates of survival and disease-free probability expressed as quartiles of γ-glutamyltransferase (GGT) variability measured as the coefficient of variance: (**a**) Myocardial infarction, (**b**) stroke, (**c**) all-cause mortality, and (**d**) cardiovascular disease-related mortality.

Kaplan–Meier curves for MI, stroke, all-cause mortality, and CVD-related mortality showed that disease-free and survival probability gradually decreased according to the higher quartiles of GGT variability, measured as GGT-CV (Figure 2). Using Cox proportional hazard models, a gradually higher risk of MI, stroke, all-cause mortality, and CVD-related mortality was observed for the higher quartiles of GGT-CV than for the lowest quartile group in all models (Table 2 and Figure S1). In GGT variability as measured using CV, the HRs for incident MI, stroke, all-cause mortality, and CVD-related mortality were 1.19 (95% CI, 1.06–1.34; *p* < 0.001), 1.20 (95% CI, 1.10–1.32; *p* < 0.001), 1.41 (95% CI, 1.33–1.51; *p* < 0.001), and 1.52 (95% CI, 1.30–1.78; *p* < 0.001), respectively, in the highest quartile of the GGT variability group, compared to the lowest quartile group after adjusting for multi-variables and mean GGT. In total cholesterol variability as measured using CV, HRs for incident MI, stroke, all-cause mortality, and CVD-related mortality were 1.22 (95% CI, 1.08–1.37; *p* = 0.003), 1.12 (95% CI, 1.02–1.23; *p* = 0.020), 1.28 (95% CI, 1.20–1.37; *p* < 0.001), and 1.22 (95% CI, 1.05–1.42; *p* = 0.022), respectively, in the highest quartile of the total cholesterol variability group, compared to the lowest quartile group after adjusting for multi-variables and mean total cholesterol (Table 2 and Figure S1). Moreover, GGT variability with SD or VIM was also identified as a meaningful predictor of mortality or CVD, even after confounding factors adjustment (Table 3 and Figure S2).

## 4. Discussion

In this large-scale cohort study with a long-term follow-up, we identified a positive association of visit-to-visit GGT variability with MI, stroke, all-cause mortality, CVD-related mortality in the younger Korean population. There was a linear relationship between GGT variability and outcome variables even after adjusting for possible confounding factors including the mean GGT level.

Several studies have documented the link between GGT and the risk of CVD and mortality. In the Framingham Study cohort that included 3451 participants, increased GGT levels were related to high risks of developing metabolic syndrome, CVD, and mortality, respectively [19]. A one-SD elevation of log-GGT increased the risk of new-onset metabolic syndrome by 26% within 20 years, increased the risk of CVD by 13%, and the risk of mortality by 26%, after adjusting for multiple variables [19]. In a prospective study of 7613 British men, GGT levels were strongly associated with all-cause mortality and death from ischemic heart disease, especially in participants with pre-existing ischemic heart disease [13]. In another study of 163,944 Austrian adults, serum GGT was independently correlated with CVD-related mortality [14]. In a meta-analysis study, Fraser et al. reported that a high GGT level was significantly associated with increased incident stroke and/or coronary heart disease [20]. Previously, we reported that serum GGT levels showed a positive linear association with MI, stroke, and all-cause mortality even after adjusting for confounding factors using the longitudinal NHIS database [15]. Interestingly, we observed that increased GGT levels were significantly related to a higher risk of 1-year mortality after an incident MI or stroke [15].

Despite numerous epidemiologic studies on the prevenient meaning of baseline GGT for adverse outcomes, few studies have reported on the longitudinal changes of GGT. In a study of middle-aged adults, Andre et al. demonstrated that an increased change in GGT levels over 3 years correlated with incident type 2 diabetes mellitus, as well as enhanced insulin resistance and increased factors of metabolic syndrome including blood pressure, triglycerides, fasting glucose, and central obesity [21]. Furthermore, one recent study reported that an increased change in GGT, even though GGT remained within the normal range, was an independent predictor of incident metabolic syndrome in middle-aged and elderly Koreans [22]. Strasak et al. demonstrated that a longitudinal increase in GGT was significantly associated with the risk of CVD mortality and all-cause mortality for a median of 10.2 years follow-up [23]. In males, an increment of GGT over 9.2 U/L in 7 years increased the risk of CVD-related mortality by 40% compared with stable GGT [23]. However, since a study by Strasak et al. started in 1985 in one province/territory of Austria, it may not sufficiently reflect the current medical environment and therapy. Furthermore, most previous studies reported the cardiometabolic disorders as surrogate markers for CVD and mortality, and all these reports showed an association between an increase in GGT levels and outcome variables. To the best of our knowledge, the association between GGT variability and incident MI, stroke, mortality, and CVD-related mortality has not previously been analyzed. In our study that comprised a nationwide population, multivariable Cox proportional hazard analyses demonstrated that visit-to-visit GGT variability was independently associated with the risk of CVD, all-cause mortality, and CVD-related mortality after adjusting for confounding factors. Recently, Kim et al. have shown that an increased risk of mortality, MI, and stroke was observed in the higher quartiles of total cholesterol variability compared with the lowest quartile in the Korean NHIS data [7]. The present study confirmed the increased HRs of mortality and CVD across the quartiles of total cholesterol variability. Interestingly, HRs of mortality and CVD across the quartiles of GGT variability were similar or even higher compared with those across the quartiles of total cholesterol variability in the same target population. This result suggests that GGT variability may be an important risk factor for mortality, CVD-related mortality, and CVD, and at least as important as total cholesterol variability within the general population.

There are still no conclusions about the association between alcohol consumption and risk of CVD events, because alcohol consumption is linked to complex mechanisms of beneficial and harmful effects [24,25]. Moderate alcohol consumption has been regarded as part of a healthy lifestyle in several studies [26,27,28] and a recent meta-analysis study showed that low or moderate alcohol consumption was related with a reduced risk of CVD [29]. However, high alcohol consumption might be considered as an increased risk for CVD [28] and mortality [27]. In the baseline characteristics, GGT variability was positively associated with the prevalence of alcohol consumption, so we adjusted for baseline alcohol consumption to evaluate the relationship between GGT variability and CVD events (or mortality).

There are several pathophysiological mechanisms regarding the effect of GGT on CVD outcomes. GGT has been suggested as a marker of pro-inflammatory actions and oxidative stress [30,31]. GGT has a catabolic action in the degradation of the antioxidant glutathione [30]. Additionally, GGT indirectly affects LDL-C oxidation in the presence of free iron [31]. The pathogenic role of oxidized LDL-C leads to aggravating plaque progression and rupture [31]. Oxidized LDL-C fluctuation, mediated through GGT variation, may contribute to exacerbating the instability of atherosclerotic plaque and induce plaque rupture. Another possibility is that participants with increased GGT variability could be accompanied with multiple comorbidities. However, our study showed a consistent relationship between GGT variability and adverse outcomes, even after adjusting for various confounding factors including conventional risk parameters and cardiometabolic disorders, which were identified using medication and medical records used as claims by physicians. The mechanism of GGT variability on cardiovascular outcomes and mortality has not been clearly elucidated and further research is needed to interpret this relationship. In cases of glucose variability, previous studies have shown that fluctuations in blood glucose levels had a greater effect on inflammatory cytokines, oxidative stress, and endothelial dysfunction than sustained hyperglycemia [32,33].

Our study has several limitations. First, even though we attempted to adjust for multiple covariates that influence mortality and/or CVD using multivariate analyses, residual or unmeasured confounding factors, such as kidney function, abdominal obesity, the amount of alcohol consumption, and the dietary patterns, remained. Second, despite excluding patients with medical diagnoses and treatment for liver disease including hepatobiliary malignancy, fibrosis and cirrhosis of liver, and viral hepatitis based on the insurance claim data, some potential confounding effects for GGT variability such as rare etiologies of liver disease, ongoing treatment, and severity of other diseases may have been included. Third, because of the inherent limitations of the observational study design, we could not determine whether a causal relationship existed between GGT variability and mortality or CVD. However, the present study also has notable strengths. The present study involved a large credible database, standardized and validated by the Korean government, with detailed information concerning medications and medical diagnosis data. Moreover, the median follow-up period of 8.4 years in this study is considered to be sufficient to assess the risk of mortality and CVD in the general population setting. Additionally, we calculated three different indicators of GGT-CV, GGT-SD, and GGT-VIM to represent the variability of GGT, and the relationships were similar and coherent.

## 5. Conclusions

This is the first study to show that visit-to-visit GGT variability is independently associated with the long-term risk of mortality and/or CVD after adjusting for established CVD risk factors including mean GGT. Because the measurements of GGT are sensitive, inexpensive, and standardized, GGT variability might be used as one predictor to stratify the risk of mortality and/or CVD. Further study is needed to replicate these findings in other ethnic groups and to reveal the mechanism of GGT variability in the pathogenesis of mortality and CVD.

## Figures and Tables

**Table 1:** Baseline characteristics of participants according to the γ-glutamyltransferase (GGT) variability (coefficient of variation).

	Q1	Q2	Q3	Q4	*p* Value
*N*	39,671	39,619	39,753	39,693	
Age (years)	56.3 ± 8.9	55.3 ± 8.5	55.5 ± 8.6	56.3 ± 8.8	<0.001
Sex (male) (*n*, %)	21,593 (54.4)	24,094 (60.8)	24,006 (60.4)	23,543 (59.3)	<0.001
Body mass index (kg/m^2^)	23.8 ± 2.8	24.0 ± 2.8	24.1 ± 2.9	24.0 ± 2.9	<0.001
Systolic BP (mmHg)	124.8 ± 15.6	125.4 ± 15.5	126.0 ± 15.7	126.7 ± 16.1	<0.001
Diastolic BP (mmHg)	77.7 ± 10.1	78.3 ± 10.1	78.6 ± 10.2	78.8 ± 10.3	<0.001
Total cholesterol (mg/dL)	198.0 ± 35.3	198.8 ± 35.7	199.4 ± 36.4	198.7 ± 38.4	<0.001
Fasting plasma glucose (mmol/L)	5.40 ± 1.31	5.45 ± 1.38	5.51 ± 1.43	5.59 ± 1.57	<0.001
Mean GGT (IU/L)	27.0 ± 25.6	32.0 ± 31.1	37.0 ± 38.6	52.9 ± 59.3	<0.001
GGT-CV (%)	10.67 ± 3.62	19.87 ± 2.36	29.09 ± 3.19	51.94 ± 18.71	<0.001
Current smoker (*n*, %)	6938 (17.5)	8269 (20.9)	8353 (21.0)	8657 (21.8)	<0.001
Alcohol consumption (*n*, %)	15,255 (38.5)	17,500 (44.2)	17,812 (44.8)	17,854 (45.0)	<0.001
Regular exercise (*n*, %)	4176 (10.5)	3940 (9.9)	3946 (9.9)	4175 (10.5)	0.002
Income (lower 10%) (*n*, %)	2932 (7.4)	3099 (7.8)	3248 (8.2)	3412 (8.6)	<0.001
Hypertension (*n*, %)	20,347 (51.3)	21,981 (55.5)	22,733 (57.2)	23,630 (59.5)	<0.001
Diabetes mellitus (*n*, %)	5164 (13.0)	6001 (15.1)	6743 (17.0)	7720 (19.4)	<0.001
Dyslipidemia (*n*, %)	10,347 (26.1)	11,153 (28.2)	11,878 (29.9)	12,607 (31.8)	<0.001

*p* value using ANOVA and Chi-square tests. Data are expressed as mean ± SD, or *n* (%). GGT, γ-glutamyltransferase; *N*, number; Q, quartile; BP, blood pressure; CV, coefficient of variation.

**Table 2:** Hazard ratios and 95% confidence intervals for myocardial infarction, stroke, all-cause mortality, and cardiovascular disease-related mortality by quartiles of γ-glutamyltransferase (GGT) variability (coefficient of variation) and total cholesterol variability (coefficient of variation).

	GGT Variability (Coefficient of Variation)				Total Cholesterol Variability (Coefficient of Variation)			
	Events (*n*)	Follow-Up Duration (Person Years)	Incidence Rate (per 1000 Person-Years)	Adjusted HR ^a^ (95% CI)	Events (*n*)	Follow-Up Duration (Person Years)	Incidence Rate (per 1000 Person-Years)	Adjusted HR ^b^ (95% CI)
Myocardial infarction								
Q1	550	326,982	1.68	1 (ref)	541	326,588	1.66	1 (ref)
Q2	538	326,090	1.65	1.00 (0.89–1.12)	564	326,328	1.73	1.09 (0.97–1.23)
Q3	638	326,175	1.96	1.14 (1.02–1.28)	563	326,019	1.73	1.04 (0.92–1.17)
Q4	710	322,430	2.20	1.19 (1.06–1.34)	768	322,743	2.38	1.22 (1.08–1.37)
*p* for trend				<0.001				0.003
Stroke								
Q1	825	326,351	2.53	1 (ref)	855	325,624	2.63	1 (ref)
Q2	803	325,372	2.47	1.04 (0.94–1.14)	780	325,806	2.39	1.00 (0.91–1.10)
Q3	923	325,408	2.84	1.14 (1.04–1.25)	847	325,337	2.60	1.01 (0.92–1.11)
Q4	1091	321,593	3.39	1.20 (1.10–1.32)	1160	321,957	3.60	1.12 (1.02–1.23)
*p* for trend				<0.001				0.020
All-cause mortality								
Q1	1550	329,166	4.71	1 (ref)	1595	328,660	4.85	1 (ref)
Q2	1544	328,170	4.70	1.08 (1.01–1.16)	1533	328,516	4.67	1.07 (1.00–1.15)
Q3	1713	328,611	5.21	1.14 (1.06–1.22)	1683	328,233	5.13	1.08 (1.01–1.16)
Q4	2500	325,122	7.69	1.41 (1.33–1.51)	2496	325,660	7.66	1.28 (1.20–1.37)
*p* for trend				<0.001				<0.001
Cardiovascular disease-related mortality								
Q1	261	329,166	0.79	1 (ref)	303	328,660	0.92	1 (ref)
Q2	287	28,170	0.87	1.22 (1.03–1.44)	280	328,516	0.85	1.05 (0.89–1.24)
Q3	356	328,611	1.08	1.43 (1.22–1.68)	285	328,233	0.87	0.98 (0.83–1.15)
Q4	434	325,122	1.33	1.52 (1.30–1.78)	470	325,660	1.44	1.22 (1.05–1.42)
*p* for trend				<0.001				0.022

^a^ Adjusted for age, sex, body mass index, alcohol consumption, smoking, regular exercise, income, diabetes mellitus, hypertension, dyslipidemia, and mean GGT. ^b^ Adjusted for age, sex, body mass index, alcohol consumption, smoking, regular exercise, income, diabetes mellitus, hypertension, dyslipidemia, and mean total cholesterol. HR, hazard ratio; GGT, γ-glutamyltransferase.

**Table 3:** Hazard ratios and 95% confidence intervals of myocardial infarction, stroke, all-cause mortality, and cardiovascular disease-related mortality by quartiles of γ-glutamyltransferase (GGT) variability (standard deviation and variability independent of the mean).

	GGT Variability (Standard Deviation)				GGT Variability (Variability Independent of the Mean)			
	Events (*n*)	Follow-Up Duration (Person Years)	Incidence Rate (per 1000 Person-Years)	Adjusted HR ^a^ (95% CI)	Events (*n*)	Follow-Up Duration (Person Years)	Incidence Rate (per 1000 Person-Years)	Adjusted HR ^a^ (95% CI)
Myocardial infarction								
Q1	446	328,049	1.36	1 (ref)	532	326,982	1.63	1 (ref)
Q2	559	327,378	1.71	1.13 (1.00–1.28)	539	327,193	1.65	1.01 (0.90–1.14)
Q3	732	324,612	2.26	1.38 (1.22–1.56)	633	325,704	1.94	1.14 (1.02-1.28)
Q4	699	321,638	2.17	1.23 (1.07–1.41)	732	321,799	2.27	1.22 (1.08–1.37)
*p* for trend				<0.001				<0.001
Stroke								
Q1	707	327,414	2.16	1 (ref)	790	326,411	2.42	1 (ref)
Q2	852	326,600	2.61	1.16 (1.05–1.28)	811	326,513	2.48	1.07 (0.97–1.18)
Q3	950	324,170	2.93	1.25 (1.13–1.39)	923	324,827	2.84	1.18 (1.07–1.30)
Q4	1133	320,540	3.53	1.44 (1.29–1.61)	1118	320,973	3.48	1.26 (1.15–1.39)
*p* for trend				<0.001				<0.001
All-cause mortality								
Q1	1388	329,799	4.21	1 (ref)	1524	329,081	4.63	1 (ref)
Q2	1582	329,472	4.80	1.11 (1.03-1.19)	1529	329,279	4.64	1.07 (0.99–1.15)
Q3	1839	327,528	5.61	1.27 (1.18–1.37)	1688	328,129	5.14	1.15 (1.07–1.23)
Q4	2498	324,270	7.70	1.53 (1.42–1.65)	2566	324,580	7.91	1.44 (1.34–1.53)
*p* for trend				<0.001				<0.001
Cardiovascular disease-related mortality								
Q1	245	329,799	0.74	1 (ref)	255	329,081	0.77	1 (ref)
Q2	305	329,472	0.93	1.24 (1.04–1.46)	292	329,279	0.89	1.24 (1.05–1.46)
Q3	373	327,528	1.14	1.54 (1.30–1.81)	349	328,129	1.06	1.44 (1.23–1.70)
Q4	415	324,270	1.28	1.74 (1.45–2.08)	442	324,580	1.36	1.60 (1.37–1.88)
*p* for trend				<0.0001				<0.0001

^a^ Adjusted for age, sex, body mass index, alcohol consumption, smoking, regular exercise, income, diabetes mellitus, hypertension, dyslipidemia, and mean GGT. GGT, γ-glutamyltransferase.

## Supplementary Materials

The following are available online at https://www.mdpi.com/2077-0383/8/6/832/s1: Table S1. Baseline characteristics of participants according to γ-glutamyltransferase (GGT) variability measured as standard deviation (SD); Table S2. Baseline characteristics of participants according to γ-glutamyltransferase (GGT) variability measured as variability independent of the mean (VIM); Figure S1. Hazard ratios and 95% confidence intervals for myocardial infarction, stroke, cardiovascular disease (CVD) related mortality, and all-cause mortality by quartiles of γ-glutamyltransferase (GGT) variability (coefficient of variation) and total cholesterol variability (coefficient of variation); Figure S2. Hazard ratios and 95% confidence intervals of myocardial infarction, stroke, cardiovascular disease (CVD) related mortality, all-cause mortality by quartiles of γ-glutamyltransferase (GGT) variability (standard deviation and variability independent of the mean).
