# Supplementary material for: γ-Glutamyltransferase Variability and the Risk of Mortality, Myocardial Infarction, and Stroke: A Nationwide Population-Based Cohort Study
Source: J Clin Med. 2019 Jun 12;8(6):832. doi: 10.3390/jcm8060832 (PMC6617005; doi:10.3390/jcm8060832)
Supplement: Supplementary file 1 [file jcm-08-00832-s001.pdf]

Table 1. Baseline characteristics of participants according to γ-glutamyltransferase (GGT) variability measured as standard deviation (SD).

	Q1	Q2	Q3	Q4	P-value
N	39639	39725	39702	39670	
Age (years)	56.3 ± 9.1	56.2 ± 8.9	55.9 ± 8.7	55.0 ± 8.2	<0.001
Sex (male) (n, %)	14154 (35.7)	20478 (51.5)	26323 (66.3)	32281 (81.4)	<0.001
Body mass index (kg/m2)	23.3 ± 2.8	23.7 ± 2.8	24.2 ± 2.8	24.5 ± 2.9	<0.001
Systolic BP (mmHg)	123.1 ± 15.7	124.7 ± 15.5	126.4 ± 15.5	128.6 ± 15.7	<0.001
Diastolic BP (mmHg)	76.4 ± 10.0	77.6 ± 10.0	78.9 ± 10.0	80.4 ± 10.3	<0.001
Total cholesterol (mg/dL)	195.7 ± 34.9	198.0 ± 35.5	200.1 ± 36.6	201.0 ± 38.6	<0.001
Fasting plasma glucose (mmol/L)	5.25 ± 1.11	5.37 ± 1.29	5.53 ± 1.43	5.79 ± 1.74	<0.001
Mean GGT (IU/L)	17.0 ± 7.1	22.1 ± 9.6	32.0 ± 15.1	77.8 ± 65.7	<0.001
GGT variability					
CV (%)	12.87 ± 6.10	22.62 ± 8.25	30.22 ± 11.41	45.87 ± 22.43	<0.001
SD (IU/L)	1.99 ± 0.75	4.41 ± 0.78	8.47 ± 1.80	35.07 ± 40.83	<0.001
VIM (%)	4.06 ± 1.78	7.43 ± 2.30	10.57 ± 3.39	18.78 ± 9.59	<0.001
Current smoker (n, %)	3902 (9.8)	6394 (16.1)	8881 (22.4)	13040 (32.9)	<0.001
Alcohol consumption (n, %)	10836 (27.3)	14289 (36.0)	18461 (46.5)	24835 (62.6)	<0.001
Regular exercise (n, %)	4297 (10.8)	4100 (10.3)	4036 (10.2)	3804 (9.6)	<0.001
Income (lower 10%) (n, %)	3268 (8.2)	3270 (8.2)	3052 (7.7)	3101 (7.8)	0.004
Hypertension (n, %)	17775 (44.8)	20637 (51.9)	23473 (59.1)	26806 (67.6)	<0.001
Diabetes mellitus (n, %)	3802 (9.6)	5196 (13.1)	6855 (17.3)	9775 (24.6)	<0.001
Dyslipidemia (n, %)	9057 (22.8)	10576 (26.6)	12232 (30.8)	14120 (35.6)	<0.001

P-value using ANOVA and Chi-square tests. Data are expressed as mean ± SD, or n (%). GGT, γ-glutamyl transferase; N, number; Q, quartile; BP, blood pressure; CV, coefficient of variation; SD, standard deviation; VIM, variability independent of the mean.

Table S2. Baseline characteristics of participants according to γ-glutamyltransferase (GGT) variability measured as variability independent of the mean (VIM)

	Q1	Q2	Q3	Q4	P-value
N	39637	39731	39685	39683	
Age (years)	56.3 ± 9.0	55.5 ± 8.6	55.6 ± 8.6	55.9 ± 8.7	<0.001
Sex (male) (n, %)	19850 (50.1)	22845 (57.5)	24285 (61.2)	26256 (66.2)	<0.001
Body mass index (kg/m2)	23.7 ± 2.8	23.9 ± 2.8	24.1 ± 2.9	24.1 ± 2.9	<0.001
Systolic BP (mmHg)	124.3 ± 15.6	125.1 ± 15.4	126.1 ± 15.7	127.3 ± 16.0	<0.001
Diastolic BP (mmHg)	77.3 ± 10.1	78.0 ± 10.0	78.6 ± 10.2	79.3 ± 10.3	<0.001
Total cholesterol (mg/dL)	197.4 ± 35.2	198.3 ± 35.7	199.7 ± 36.3	199.5 ± 38.5	<0.001
Fasting plasma glucose (mmol/L)	5.36 ± 1.26	5.42 ± 1.33	5.51 ± 1.42	5.66 ± 1.65	<0.001
Mean GGT (IU/L)	23.7 ± 20.0	27.9 ± 22.2	35.2 ± 30.6	62.1 ± 65.4	<0.001
GGT variability					
CV (%)	10.78 ± 3.77	20.11 ± 3.04	29.28 ± 4.47	51.41 ± 19.19	<0.001
SD (IU/L)	2.46 ± 1.76	5.30 ± 3.29	9.60 ± 6.64	32.58 ± 41.83	<0.001
VIM (%)	3.60 ± 1.24	6.87 ± 0.86	10.33 ± 1.23	20.04 ± 8.70	<0.001
Current smoker (n, %)	6164 (15.6)	7587 (19.1)	8447 (21.3)	10019 (25.2)	<0.001
Alcohol consumption (n, %)	14130 (35.6)	16375 (41.2)	17929 (45.2)	19987 (50.4)	<0.001
Regular exercise (n, %)	4207 (10.6)	4045 (10.2)	3867 (9.7)	4118 (10.4)	<0.001
Income (lower 10%) (n, %)	3024 (7.6)	3101 (7.8)	3239 (8.2)	3327 (8.4)	<0.001
Hypertension (n, %)	19679 (49.6)	21526 (54.2)	22750 (57.3)	24736 (62.3)	<0.001
Diabetes mellitus (n, %)	4771 (12.0)	5723 (14.4)	6700 (16.9)	8434 (21.3)	<0.001
Dyslipidemia (n, %)	10010 (25.3)	10856 (27.3)	11923 (30.0)	13196 (33.3)	<0.001

P-value using ANOVA and Chi-square tests. Data are expressed as mean ± SD, or n (%). GGT, γ-glutamyl transferase; N, number; Q, quartile; BP, blood pressure; CV, coefficient of variation; SD, standard deviation; VIM, variability independent of the mean.

Figure S1. Hazard ratios and 95% confidence intervals for myocardial infarction, stroke, all-cause mortality, and cardiovascular disease related mortality by quartiles of γ-glutamyltransferase (GGT) variability (coefficient of variation) and total cholesterol variability (coefficient of variation).

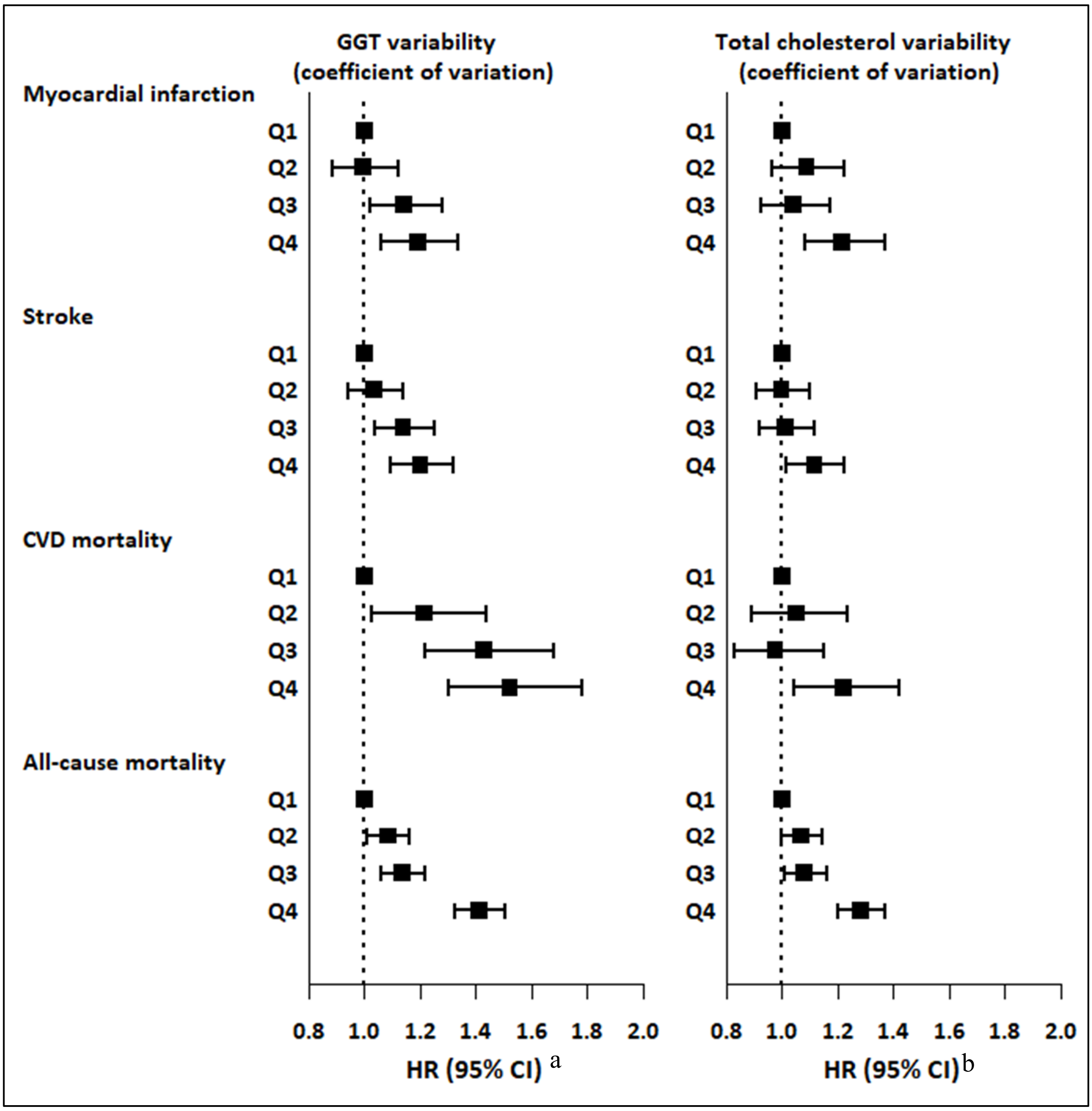

a Adjusted for age, sex, body mass index, alcohol consumption, smoking, regular exercise, income, diabetes mellitus, hypertension, dyslipidaemia, and mean GGT. b Adjusted for age, sex, body mass index, alcohol consumption, smoking, regular exercise, income, diabetes mellitus, hypertension, dyslipidaemia, and mean total cholesterol. GGT, γ-glutamyl transferase; CVD, cardiovascular disease

Figure S2. Hazard ratios and 95% confidence intervals of myocardial infarction, stroke, all-cause mortality, and cardiovascular disease related mortality by quartiles of γ-glutamyltransferase (GGT) variability (standard deviation and variability independent of the mean).

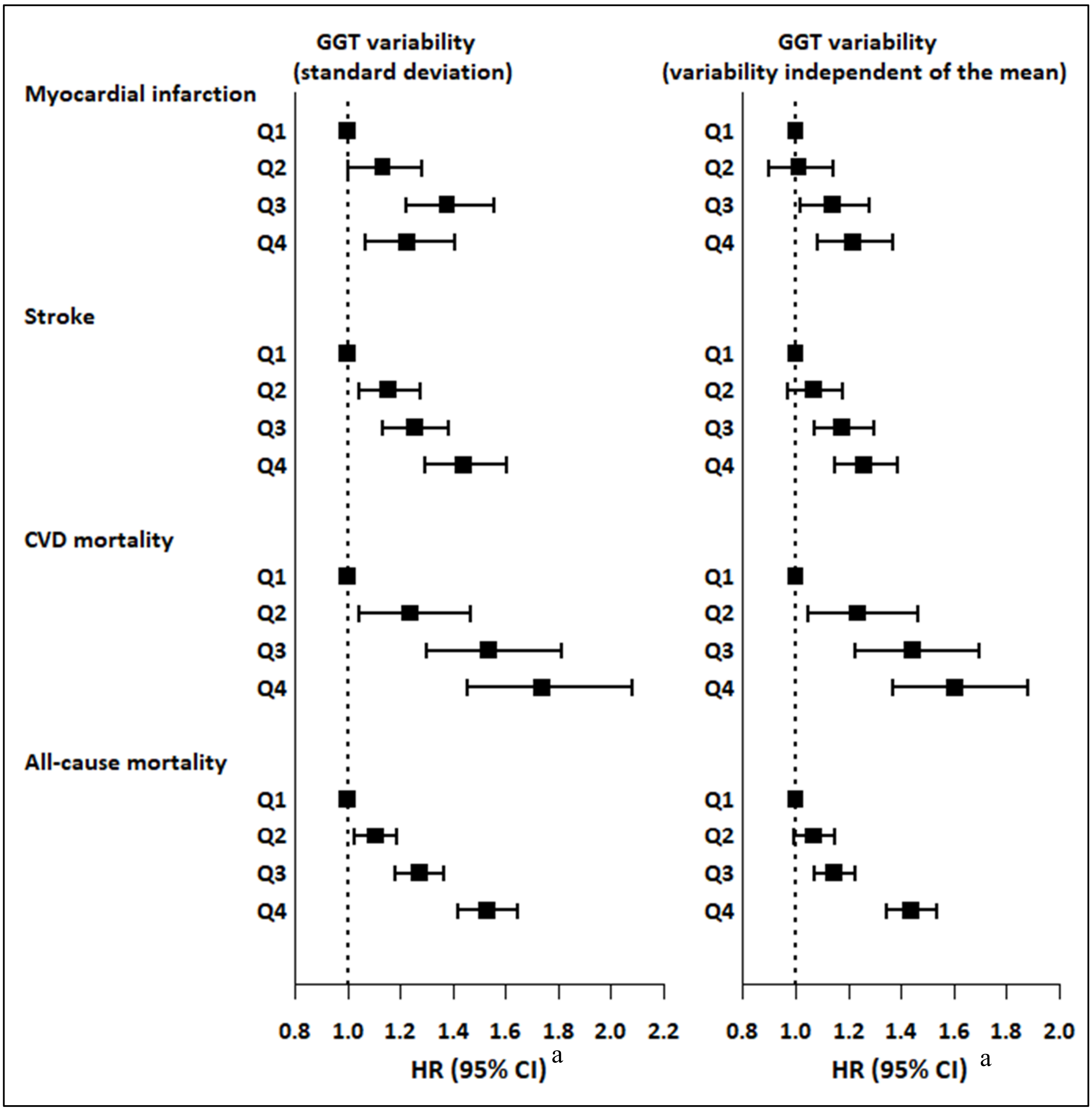

a Adjusted for age, sex, body mass index, alcohol consumption, smoking, regular exercise, income, diabetes mellitus, hypertension, dyslipidaemia, and mean GGT. GGT, γ-glutamyl transferase; CVD, cardiovascular disease
